# Research Trends in Individuals at High Risk for Psychosis: A Bibliometric Analysis

**DOI:** 10.3389/fpsyt.2022.853296

**Published:** 2022-04-29

**Authors:** Tae Young Lee, Soo Sang Lee, Byoung-gyu Gong, Jun Soo Kwon

**Affiliations:** ^1^Department of Psychiatry, Pusan National University Yangsan Hospital, Yangsan-si, South Korea; ^2^Research Institute for Convergence of Biomedical Science and Technology, Pusan National University Yangsan Hospital, Yangsan-si, South Korea; ^3^Department of Library Information Archives Studies, Pusan National University, Pusan, South Korea; ^4^Sorenson Impact Center, University of Utah, Salt Lake City, UT, United States; ^5^Department of Psychiatry, Seoul National University College of Medicine, Seoul, South Korea; ^6^Department of Brain and Cognitive Sciences, Seoul National University College of National Sciences, Seoul, South Korea

**Keywords:** bibliometric analysis, collaborative study, schizophrenia, clinical high risk for psychosis, research network, research trends

## Abstract

The study of clinical high risk for psychosis (CHR-P) has progressed rapidly over the last decades and has developed into a significant branch of schizophrenia research. Organizing the information about this rapidly growing subject through bibliometric analysis enables us to gain a better understanding of current research trends and future directions to be pursued. Electronic searches from January 1991 to December 2020 yielded 5,601 studies, and included 1,637 original articles. After processing the data, we were able to determine that this field has grown significantly in a short period of time. It has been confirmed that researchers, institutions, and countries are collaborating closely to conduct research; moreover, these networks are becoming increasingly complex over time. Additionally, there was a shift over time in the focus of the research subject from the prodrome, recognition, prevention, diagnosis to cognition, neuroimaging, neurotransmitters, cannabis, and stigma. We should aim for collaborative studies in which various countries participate, thus covering a wider range of races and cultures than would be covered by only a few countries.

## Introduction

Many patients with schizophrenia, although not in all patients, experience a period of attenuated or transient psychotic symptoms and functional decline preceded by the onset of psychosis, which is referred to as the prodromal stage (1, 2). Based on the achievements of pioneers in this field, interest in in the prevention of schizophrenia increased during a pivotal period (3–5). In the beginning, research was conducted on offspring or relatives of patients with schizophrenia, who were regarded as a genetically high-risk group, but the study’s challenges were aggravated by the long follow-up duration and low incidence rate (6). The concept of ultra-high risk (UHR) or clinical high risk for psychosis (CHR-P) as a prospective aspect, which has the potential to transition into psychosis in the future, was proposed, and diagnostic instruments were developed (7–10). Large cohort studies have begun in Australia, North America, the United Kingdom, and Germany (2, 11–13). Approximately 35% of CHR-P patients develop psychosis after many years of follow-up; even among non-converters, the rate of complete remission is low, and many continue to exhibit functional impairment (14–16). Even before the commencement of psychosis, patients exhibit deterioration in cognitive and social cognitive function (17, 18). Cortical thinning, aberrant thalamocortical connectivity and abnormalities in event-related potentials have also been observed in CHR-P (19–21). Alterations in the dopamine-glutamate system have also been intensely examined in recent years (22, 23). Antipsychotic medications have not been proven to prevent the onset of psychosis in CHR-P, and it remains controversial whether cognitive–behavioral therapy or omega-3 fatty acid supplements could be an effective treatment (24–27). Given the heterogeneity of psychosis, it is assumed that patients with a variety of psychopathologies are still classified as CHR-P, and this situation could be linked to the difficulty of developing effective predictions (28–30). To address this, many efforts have been undertaken to develop a personalized prediction model that takes individual characteristics into account (31–34). However, the recruiting subjects through expansion of outreach and the decreasing incidence of psychosis make obtaining a sufficient sample size for robust analysis and external validation of prediction model troubling (35, 36). Recently, several collaborative studies have been initiated to overcome these hurdles, and these large-scale biomarker studies are expected to shed light on the present understanding of the pathophysiology of schizophrenia and the discovery of effective treatments, such as HARMONY, NAPLS, ProNET, PRONIA, and PSYSCAN (37–41). The study of CHR-P has progressed rapidly over the last decades and has developed into a significant branch of schizophrenia research. Thus, organizing information about this rapidly growing subject through bibliometric analysis helps us to gain a better understanding of current research trends and future directions to be pursued.

Bibliometric analysis is a research method for systemic literature review of a specific field, topic, and discipline in which research trends with authors, journal, keyword, cited reference, institution, and country-related indicators are quantified. Research at a more advanced level attempts to broadly map out a research area representing the field using a bibliographic network, such as citation, co-occurrence, and collaboration networks (42). This visualization method, sometimes called science mapping, is a powerful way to provide a topological description of the fields with quantitative indicators. The connection between documents, journals, or authors represented by bibliographic networks reveals power dynamics and knowledge hierarchy in the field. With this information, researchers could detect a latent community structure through patterned networks, discover a hidden structure of an intellectual community and envision its evolution.

In the present study, we examine which authors, institutions, and nations contributed to high-risk research conducted during the last decades and explore how they are connected and change over time based on bibliometric analysis. Additionally, we evaluate research topics in high-risk research through keyword analysis and assess how these topics are merged and related over time.

## Materials and Methods

A systematic search strategy identified relevant studies. Two independent researchers (T. Y. L. and N. S. K.) conducted a two-step literature search. First, a literature search using PubMed and Web of Science was performed to identify relevant articles from January 1991 to December 2020. The following keywords, including their synonyms and combinations, were used as search terms: “psychosis risk,” “clinical-high risk,” “UHR,” “at-risk mental states,” “basic symptoms,” and “prodromal psychosis,” In the second step, the reference lists of the published reviews and studies were manually checked to identify additional relevant publications. We deleted duplicate literature from the full list, then only original research papers were adopted, excluding gray literature or non-original papers like reviews, meta-analyses, letters, editorials, and conference abstracts.

We analyzed these refined bibliometric data using the R v4.2 to process large-sized bibliometric and textual data efficiently. The process included the following steps. First, we performed an explorative, descriptive analysis by converting raw bibliometric data into interpretable textual data, showing how many authors and journals have been involved and how many publications have been produced thus far in this field. Second, we identified the most influential publications, authors, journals, countries, and institutions based on citation number and publication count overtime *via* Web of Science Core collection. Third, we mapped out collaboration networks between countries and institutions based on the co-occurrence of multiple country or institution names in the publications. We used the Louvain method for community detection. The Louvain method is based on the modularity score, the difference between the actual edge count in the cluster, and the item’s random chance of being in the cluster (43). The Louvain method is designed to optimize the modularity score through the iterative process of moving one vertex at a time from one group to another and calculating the score at every step. Fourth and last, we traced the thematic evolution of research topics over time, examining how the thematic cluster identified in previous time T evolved in later time T + 1. We lemmatized the keywords to standardize differential terms, removing the suffix of derivative terms. Additionally, we removed specific terms used as the search keyword because these terms can be too dominant and mask the entire keyword network. Then, we calculated the inclusion index and used it to create plots showing thematic evolution (44). The inclusion index is based on the number of shared keywords between clusters in two different time periods. The lines between clusters indicate this inclusion index score, and its thickness implies the shared number of keywords between them.

## Results

### Papers

Electronic searches yielded 5,601 studies, and 1,637 studies were included in bibliometric analysis. Some studies were excluded because they were included erroneously (*N* = 143), were non-English papers (*N* = 160), were non-original research papers (*N* = 2101), were duplicates (*N* = 129), had different ranges of publication years (*N* = 339), included non-psychiatric illnesses using the high-risk concept (*N* = 102), were animal studies (*N* = 20), or were about different topics (*N* = 970).

**FIGURE 1:**
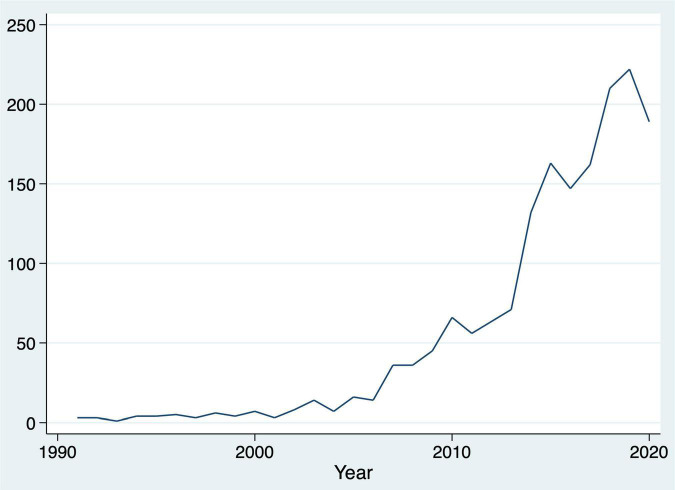
Number of publications per year.

The publications spanned from 1991 to 2020. Figure 1 shows the annual publication number from 1990 to 2000, and it depicts a surge in the research in this field from approximately 2014, reaching more than 200 annual publications in 2018. There were 259 journals in which at least one relevant study was published (Supplementary Table 1). Among them, the top 7 journals, Schizophrenia Research, Early Intervention in Psychiatry, Schizophrenia Bulletin, Psychiatry Research, Psychological Medicine, Frontiers in Psychiatry, and European Psychiatry, published more than half of the total number of papers. Relevant papers were cited 51,925 times in total. Most of the papers, except for 57, were cited more than once, and each was cited an average of 31.7 times. Among them, papers in the top 5 journals, Schizophrenia Research, Schizophrenia Bulletin, Archives of General Psychiatry, Psychological Medicine, and Biological Psychiatry, accounted for half of the total. The top 100 most cited papers are listed in the Supplementary Table 2.

### Authors

**FIGURE 2:**
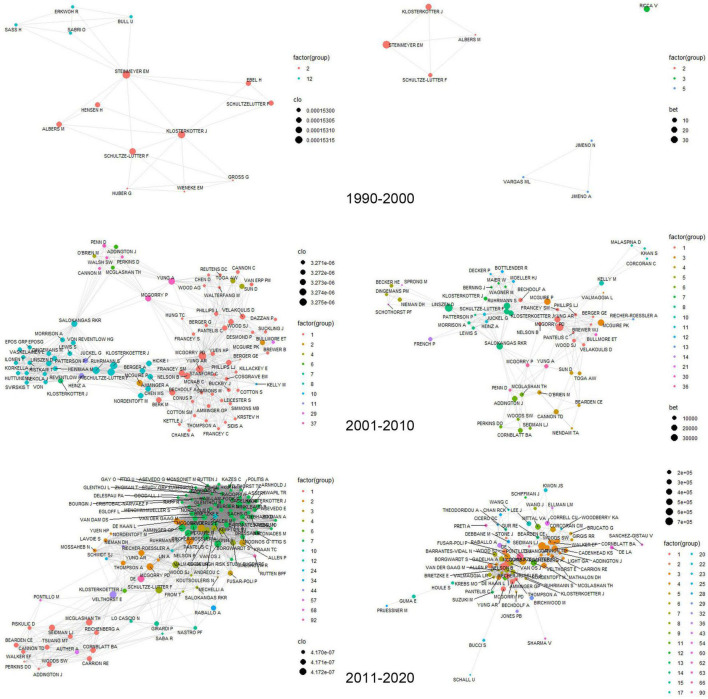
The collaboration network among the authors over a decade. The figure on the left is the closeness centrality and the figure on the right is the betweenness centrality.

The number of unique authors in the collected papers was 5,281. The average number of publications by each author was 2.4, and the median number was 1. Most of the authors produced documents through collaborative teamwork, while only a marginally small number of authors, 22, published alone. The average number of citations by authors was 69.2, and the median number was 13. The author with 7,916 total citations ranked first, and articles by 1,380 authors had not been cited yet. The co-authorship networks were intricately connected regardless of institution and country, and the main results are described below. We analyzed the influence of authors in this field through the number of published papers, the number of citations, and the network index of authors’ co-authorship (Figure 2 and Supplementary Table 3).

### Institutions

**FIGURE 3:**
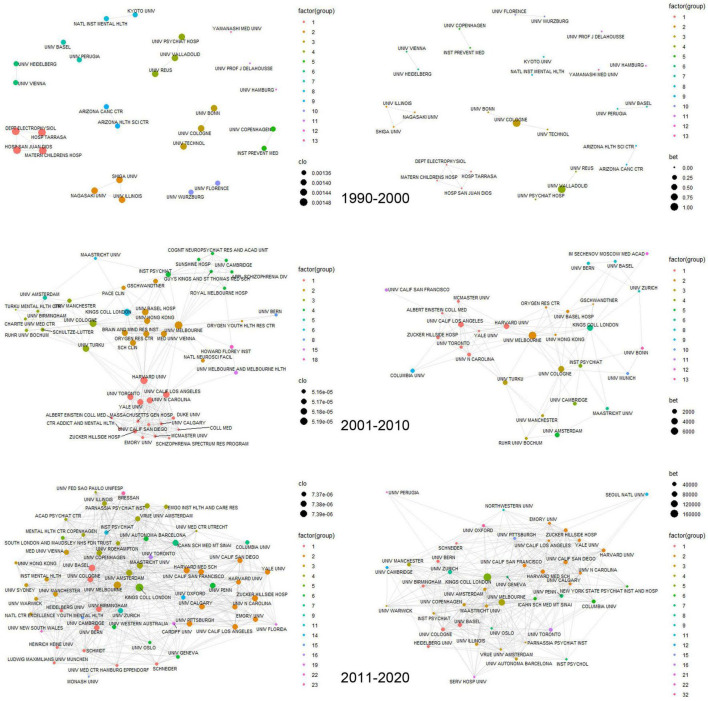
The collaboration network among the research institutions over a decade. The figure on the left is the closeness centrality and the figure on the right is the betweenness centrality.

A total of 1,573 institutions were associated with more than one publication. The average number of publications by each institution was 4.3, and the median number was 1. The institution that has published 187 papers was the first rank, and 893 institutions were involved in only one paper. The papers were cited 186,972 times in total, while papers from 371 institutions have not yet been cited. The average number of citations by each institution was 118.9, and the median number was 16. Figure 3 and Supplementary Table 4 represents the collaboration network among the research institutions.

### Countries

**FIGURE 4:**
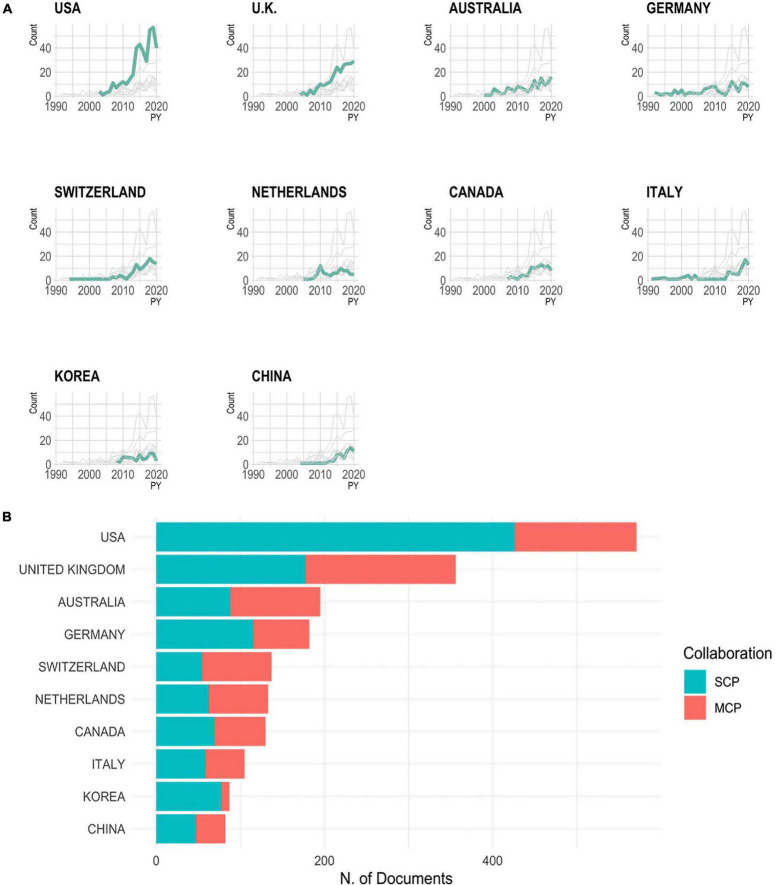
Changes in the number of publications in each country. SCP: single country publication, MCP: multiple country publication.

A country in this analysis is the country of the corresponding author’s institution when each paper was published. Figure 4 and Supplementary Table 5 portrays the country publication data in detail. Figure 4A represents publication counts across time from 1990 to 2020. Figure 4B shows the country publication data categorized into two-, single- and multiple-country publications.

### Keywords

**FIGURE 5:**
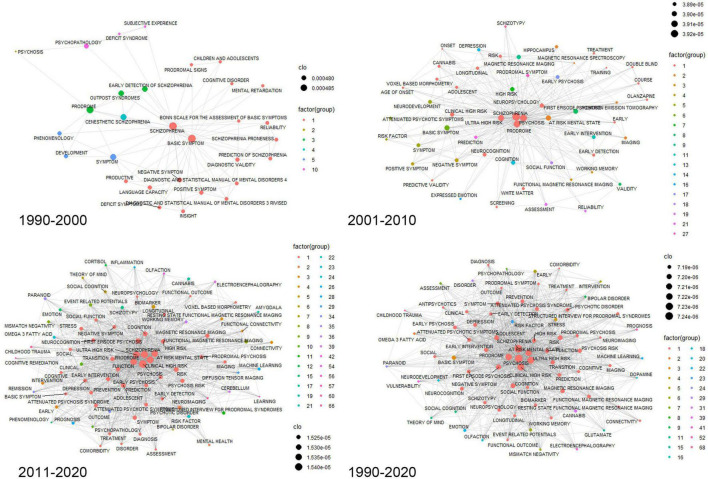
Keyword co-occurrence network. Centrality measure used is closeness centrality.

A total of 8,478 author-suggested keywords were extracted from all publications, of which 2,511 keywords were screened to exclude duplicates prior to data cleaning. We analyzed 12,030 keywords extracted from 1,637 documents. In Figure 5, the results of closeness centrality utilizing the retrieved keywords are showed by decade and total period (Supplementary Table 6). With the exception of “prodrome” and “at-risk mental state,” which refer to the CHR-P, keywords such as “cognition,” “transition,” “MRI,” “function,” “early intervention,” and “depression” were found to be the most important top 10 keywords throughout the period in terms of closeness centrality.

## Discussion

In the present study, a bibliometric analysis was implemented to examine the current status and trends in the study of CHR-P. Analysis of the processed data revealed that this field has grown significantly in a short period of time. It has been confirmed that researchers, institutions, and countries are collaborating closely to conduct research, and these networks are becoming increasingly complex over time. Additionally, there was a shift over time in the focus of the research subject from the prodrome, recognition, prevention, diagnosis to cognition, neuroimaging, neurotransmitters, cannabis, and stigma.

Among institutions researching CHR-P, King’s College London and University of Melbourne published the largest number of papers. In the case of the institutions in NAPLS consortium, one of the largest cohorts in CHR-P research, statistics are dispersed across the consortium’s member institutions since the site responsible for the study varied according to the research topic. This reflects the method of this study, which counts the number of citations based on the corresponding author. On the other hand, the University of Melbourne had the highest citation numbers. This result appeared to be because this site is one of the institutions with the longest research period regarding the term “CHR-P.” Apart from prominent institutions in North America and Europe, institutions from other continents, such as Shanghai University, Seoul National University, University of São Paulo, and Yonsei University stood out in terms of research activity, while other institutions were not yet immersed in research (45–48). In this result, however, the names of various affiliated institutions within a university or hospital were counted separately. Thus, whether a single institution name is used or the names of the various or subsidiary institutions on the site are used differently would affect the actual influence of the institution that was noted. Each institution is inextricably linked, and the network continues to develop in size and density over time. Early phase of the high-risk research, German sites centered on basic symptoms opened the field (49). The networks were developed mostly in Europe and North America separately, and it is presumed that the employment of the Comprehensive Assessment of At-Risk Mental States (CAARMS) and the Structured Interview of Psychosis-risk Syndromes (SIPS), respectively, which are diagnostic instruments for high-risk groups, had an effect (50). The network scale will be progressively enlarged, particularly for biological studies involving brain imaging or blood. Additionally, due to the nature of external validation in model development, a broader network investigation involving consortium collaboration will be more required in the future (38, 50–52).

In the findings from countries, the United Statesand United Kingdom. started dramatically increasing the number of relevant research articles produced in approximately 2010, outperforming other countries with a considerable margin, while relevant publication in the rest of the countries remained relatively flat. Italy, Switzerland, and China are emerging players in this field. The United Kingdom, Australia, Switzerland, Netherlands, and Canada have a relatively high proportion of multiple-country publications, accounting for almost half of the total publications. Meanwhile, the United States, Germany, and Korea have a relatively low proportion of multiple-country publications. In the case of the United States, NAPLS is a large-scale, multi-institution domestic study, and Germany has long had a domestic consortium focusing on basic symptoms. In the case of Korea, the reason is presumed to be from the non-Latin linguistic region. This indicates that the former group of countries tend to conduct intercountry collaborative research, while the latter group of countries tend to rely on intra-country collaboration networks. However, as networks become more connected over time, this distinction is increasingly being overcome through large-scale collaborative studies such as ENIGMA, HARMONY, ProNET, PRONIA, and PSYSCAN (41). Although these collaboration studies that are being promoted recently were not well revealed in the results of this study based on the number of citations, these are expected to solve the limitation of an insufficient sample size of the converters that have been raised so far. In addition, large-scale collaboration studies linking multiple continents will aid in the study of various pathophysiology, such as the differences of race or ethnicity on the transition to psychosis.

Distinct patterns of symptom expression, stigma, and care-seeking strategies were noted depending on cultural differences; social standards for weird behavior or deviant beliefs also differed (53–55). Pertinently, drug responses and genes differ according to race and ethnicity (56). These issues are crucial for understanding the pathophysiology of schizophrenia. Therefore, we should aim for collaborative studies in which various countries participate, thus covering a wider range of races and cultures than would be covered by only a few countries. Moreover, increased diversity within scientific research organizations should be considered (57).

Keyword analysis is the cherry on top of bibliographic analysis, but it is also a more complex and arbitrary aspect to deal with. To minimize researchers’ bias, we only performed minimal processing in the data cleaning for keyword analysis. While this strategy helps prevent arbitrary distortion, it has a drawback in that the actual influence of keywords with a myriad of derivatives and hierarchies is disregarded. For example, combining keywords such as P3, P3a, and P300 into one element helps to emphasize the prominence of the issue that many researchers repeatedly address; however, if other keywords with similar forms cannot be treated in the same manner, keywords preprocessing will distort the value of the study topic. As a result, the keyword’s weight would be fragmented by one-third. Additionally, this analysis has a characteristic that focuses more on the frequency than the impact of the subject. For example, while 22q11.2 deletion syndrome has recently been recognized as an intriguing research model for schizophrenia (58, 59), its influence on keywork analysis may be underestimated due to the limited number of researchers capable of conducting the study directly in comparison to its importance in the field. In this study, we found that the most frequently used keywords in this field shifted from “prodrome,” “early recognition,” “primary prevention,” “diagnostic validity,” “reliability,” and “antipsychotics” in the first decade to “cognition,” “magnetic resonance imaging,” “social function,” “hippocampus,” “prefrontal cortex,” “serotonin,” “schizotypy,” and “positron emission tomography” in the next decade. Finally, the most frequently used keywords shifted to “transition,” “stress,” “functional magnetic resonance imaging,” “diffusion tensor imaging,” “event-related potential,” “cannabis,” “functional connectivity,” “dopamine,” and “stigma” in the last decade in this field. Notably, research in this field is evolving from symptoms and diagnosis to cognitive function and finally to biological topics. This trend indicates that research utilizing biomarkers to predict the onset of psychosis is being undertaken in earnest. Indeed, CHR-P, along with psychotic disorders, is the research field where the largest number of prognostic studies are being performed in psychiatry (60).

This study has several limitations. First, we included only original research papers and excluded non-original papers such as meta-analyses, reviews, letters. Although we evaluated the research trends based original research publications, we also recognize that non-original research, such as meta-analysis or editorials, can be critical in alerting people about new facts or generating interest in research. As a result, it is possible that it was overlooked in our results. Second, we excluded papers written in languages other than English. This could be interpreted as a reduction in the impact of research to non-English speaking countries. Third, studies with different criteria were lumped together. Even if the study is not a CHR-P study utilizing instruments such as CAARMS and SIPS, follow-up studies with relatives of schizophrenia or with psychotic-like experiences could be included. Due to the heterogeneity of samples, it will be necessary to analyze only studies with more stringent criteria in the future.

## Data Availability Statement

The original contributions presented in the study are included in the article/Supplementary Material, further inquiries can be directed to the corresponding author.

## Author Contributions

TL and JK were responsible for the design of the whole study and wrote the protocol. TL wrote the manuscript. SL and B-GG performed the statistical analysis. All authors listed have made a substantial contribution to the work and approved it for publication.

## Conflict of Interest

The authors declare that the research was conducted in the absence of any commercial or financial relationships that could be construed as a potential conflict of interest.

## Publisher’s Note

All claims expressed in this article are solely those of the authors and do not necessarily represent those of their affiliated organizations, or those of the publisher, the editors and the reviewers. Any product that may be evaluated in this article, or claim that may be made by its manufacturer, is not guaranteed or endorsed by the publisher.
